# A Treatise on Nervous and Mental Diseases, for Students and Practitioners of Medicine

**Published:** 1893-03

**Authors:** 


A Treatise on Nervous and Mental Diseases, for Students and Practitioners of Medicine. By Landon Carter Gray, M. D., Professor of Nervous and Mental Diseases, in the New York Polyclinic, etc., with 168 illustrations, 8vo, pp. 686, cloth. Philadelphia: Lea Brothers & Co. 1893.

This volume is the result of many years' experience in the neurological field, and is written with the firm conviction that a book was needed which considered nervous and mental diseases chiefly from the therapeutic standpoint. The physician, well read in this subject, will be surprised on reading this work that many clinical points in the several diseases have been omitted. This has not been carelessness, but intentional. The author says: "A teacher must know what to omit, as the force and permanence of his teachings will depend upon his strict avoidance of the non-essentials." In the discussion of what to omit, men will naturally differ, for therein is the element of personal equation.

We think that the excellent chapter on Epilepsy would have been more valuable had the author told the reader just what was meant by an aura. All that he says on the subject is: "There may be other sensations called auræ."

In the discussion of migraine, we hoped that the author would discuss ocular defects as an etiological factor, and the correction of these defects as a therapeutic measure. But he does not even mention the fact that it is possible for a case of migraine to be aggravated by any ocular defect. The explanation of this is found in his belief that all cases are hereditary. It also explains the statement he makes: "I have never known one being cured."

In the article on Chorea we should have expected to see some mention of post-choreic paralysis, and also of the limp or paralytic chorea. But these are simply the omissions which are dictated by selection of the non-essentials, and we simply record our regret that what we have held to be so highly important as the aura of epilepsy, ocular deficiencies in migraine, and the paralytic phenomena in chorea, should not find place in this valuable volume.

The arrangement has been most admirable, the style very clear. The reader will never be in doubt as to what is meant when he reads a chapter in Gray.

The articles on the Spinal Cord are very clear, and clinical types are ably presented. The various methods of treatment are given their full value, even though the author has not full faith, as, for instance, his report of suspension in locomotor ataxia.

We were glad to notice the prominent place he accords hypnotism in this book, for we believe with the author, that in it we have an agent of value.

In his views of treatment and prognosis the author is apt to take more hopeful views than we usually see in works on nervous disease. This is most gratifying, as it will tend to arrest the tendency, in so many men, of thinking their duty accomplished when they have made a diagnosis.

There is a marked exception to this in his view of Raynaud's disease. He here is flatly opposed to the opinion of Dana, in his text-book of nervous disease, who says "the cases usually get well." He also differs from Osler, who, in his masterly work, says of this disease: "Attacks of this sort may recur for years."

"Apart from this unpleasant symptom the general health may be very good."

Gray takes the following gloomy view: "The prognosis of Raynaud's disease and erythromelalgia are unmitigatedly bad." "The treatment of Raynaud's disease must be amputation as speedily as possible, before the strength of the patient is exhausted and before sepsis sets in."

We think that, as the author's views on this subject differ so radically from those of all other writers, that in a second edition some hint should be given that in this view he stands alone.

Part III. is devoted to a consideration of mental diseases. This consists of less than 100 pages, and, hence, cannot be considered in the light of a treatise on insanity. On the contrary, the various forms are merely sketched. It bears the evidence of being hurriedly completed, or else of the author's feeling the limitation of space.

He mentions a will case in which the testator had illusions of smell, and yet protests against the man's testamentary capacity, without giving any other reason for it than hallucination of smell. The reader should not be left to infer that such a symptom, unsupported by other evidences of insanity, would or should affect the power of making a will.

The chapter on Melancholia is very complete, and conveys a remarkably good idea of the condition in a short space. We are in doubt, however, as to other observers seeing so large a proportion of cases with post-cervical ache.

The chapter on Mania is noticeably incomplete, as also are the chapters on Hebephrenia and Furor Transitorius. We think that a clearer idea of the various forms would be given were there some illustrative cases. The author is decided in his opposition to asylum treatment for the curable insane. In order to follow this theory we must become far better prognosticians than we are at present.

The value of this book is greatly increased by the beauty and original illustrations. The bibliography is of great aid in reference work, and a complete glossary will do much to aid the student in his understanding of neurological terms.

While we have pointed out wherein we differ from the author in our estimate of the non-essentials, as a whole we cannot but believe that it will prove a most useful addition to our literature.

